# Burst firing versus synchrony in a gap junction connected olfactory bulb mitral cell network model

**DOI:** 10.3389/fncom.2012.00075

**Published:** 2012-09-27

**Authors:** Simon O'Connor, Kamilla Angelo, Tim J. C. Jacob

**Affiliations:** ^1^School of Biosciences, Cardiff UniversityCardiff, UK; ^2^Department of Neuroscience, Physiology, and Pharmacology, University College LondonLondon, UK

**Keywords:** olfactory bulb model, mitral cells, oscillation, synchronization, passive parameters, burst firing

## Abstract

A key player in olfactory processing is the olfactory bulb (OB) mitral cell (MC). We have used dual whole-cell patch-clamp recordings from the apical dendrite and cell soma of MCs to develop a passive compartmental model based on detailed morphological reconstructions of the same cells. Matching the model to traces recorded in experiments we find: *C*_*m*_ = 1.91 ± 0.20 μF cm^−2^, *R*_*m*_ = 3547 ± 1934 Ω cm^2^ and *R*_*i*_ = 173 ± 99 Ω cm. We have constructed a six MC gap-junction (GJ) network model of morphologically accurate MCs. These passive parameters (PPs) were then incorporated into the model with Na^+^, Kdr, and KA conductances and GJs from Migliore et al. (2005). The GJs were placed in the apical dendrite tuft (ADT) and their conductance adjusted to give a coupling ratio between MCs consistent with experimental findings (~0.04). Firing at ~50 Hz was induced in all six MCs with continuous current injections (0.05–0.07 nA) at 20 locations to the ADT of two of the MCs. It was found that MCs in the network synchronized better when they shared identical PPs rather than using their own PPs for the fit suggesting that the OB may have populations of MCs tuned for synchrony. The addition of calcium-activated potassium channels (iKCa) and L-type calcium channels (iCa(L)) (Bhalla and Bower, 1993) to the model enabled MCs to generate burst firing. However, the GJ coupling was no longer sufficient to synchronize firing. When cells were stimulated by a continuous current injection there was an initial period of asynchronous burst firing followed after ~120 ms by synchronous repetitive firing. This occurred as intracellular calcium fell due to reduced iCa(L) activity. The kinetics of one of the iCa(L) gate variables, which had a long activation time constant (τ ~ range 18–150 ms), was responsible for this fall in iCa(L). The model makes predictions about the nature of the kinetics of the calcium current that will need experimental verification.

## Introduction

The olfactory bulb (OB) represents an opportunity to access, in isolation, the first level of processing of sensory input (Shepherd and Greer, 1998). The mitral cells (MCs)^1^ are the principle output cells of the OB, and in this role they receive the sensory input from the olfactory receptor neurons and output the processed signal to the olfactory cortex. An understanding of how input and output are related in the MC is therefore key to understanding the first level of processing of sensory input in the olfactory system.

The neuropharmacological influences on the MCs are complex (reviewed in O'Connor and Jacob, 2008). Computational modeling has been used to understand these interactions at a macro level (e.g., Linster and Cleland, 2009, 2010; Marella and Ermentrout, 2010), however, there is also a need to understand the biophysics of these interactions at a more detailed scale and to consider how the MCs integrate input and interact in local microcircuits. To do this strategies have to be developed to constrain the variables. As a precedent for this approach there is the pioneering work of (Bhalla and Bower, 1993) followed by a sequence of more targeted studies (Shen et al., 1999; Chen et al., 2002; Migliore et al., 2005, 2007, 2010; Migliore and Shepherd, 2008; McTavish et al., 2012). The Bhalla and Bower model (Bhalla and Bower, 1993) was created from parameters borrowed from similar cells in other brain regions and parameters were then adapted by fitting to OB recordings. The other group of studies aimed at a more tightly constrained approach to model correctly the shift of the action potential from axon to dendrites (Shen et al., 1999). This was followed by adapting the model to look at intercellular synchronization via gap junctions, for which the morphology was modeled as a simple canonical structure (Migliore et al., 2005). There followed a series of models that investigated the interactions of MC lateral dendrites in mitral-granule cell networks. These are cell networks with a complex morphology.

In a range of studies in other brain regions, it has been shown that cell morphology as well as conductance distribution has a strong effect on action potential initiation and intrinsic firing patterns (Kim and Connors, 1993; Mainen and Sejnowski, 1996; Vetter et al., 2001; Schaefer et al., 2003). It is therefore timely to bring a more realistic morphology to the understanding of action potential initiation and firing patterns of the MC. This was the starting point for a new model targeting the biophysics of MCs.

Estimation of passive parameters (PPs) from somatic recordings has had a long history (including Rall, 1959; Barrett and Crill, 1974; Clements and Redman, 1989; Major et al., 1994; Thurbon et al., 1998; Perreault and Raastad, 2006). The use of two site electrophysiology with an accurate morphological reconstruction to fit PPs is more recent and has been applied to neocortical pyramidal neurons (Stuart and Spruston, 1998; Golding et al., 2005) and Purkinje cells (Roth and Häusser, 2001). Here we apply the same method to the MCs of the rat OB. Shen et al. (1999) carried out direct fitting of seven PPs to the first 4 ms after the onset of a current injection in a two site injection/recording protocol. These PPs include dendritic diameters in a simple canonical model. Here we apply MC recordings using a two site recording protocol for direct fitting of three PPs to current injection transients using accurate 3D reconstructions of the actual cell from which the transients were recorded.

The OB consists of multiple processing channels, with each channel dedicated to integrating the input from an olfactory receptor neuron expressing a single olfactory receptor protein (O'Connor and Jacob, 2008). The individual channels contain populations of MCs that are coupled by gap junctions between their apical dendrite tufts (ADTs) in a single glomerulus. The correlation in electrical activity between pairs of MCs in these intra-glomerular populations has been studied in experimental recordings (Schoppa and Westbrook, 2001, 2002; Margrie and Schaefer, 2003) and modeling (Migliore et al., 2005). To incorporate these population properties of the MC, we extended our modeling to a small network of MCs coupled at the ADT by gap-junctions (GJs). For this we used the Migliore model (Migliore et al., 2005) Na, K_dr_, and K_A_ channel mechanisms. Migliore and co-workers in their paired MC synchronization tests deliberately used this simple set of channel mechanisms and evoked tonic firing by a continuous injection current. This was to avoid complicating sources of periodicity that might influence the synchronization of the paired MCs. It is the synchronized firing of MCs that generates the experimentally observed oscillations in the OB.

Oscillations occur at a wide range of frequencies in the OB. There are reports of gamma (30–80 Hz; Adrian, 1950; Bressler and Freeman, 1980), beta (15–30 Hz; Kay and Freeman, 1998), theta (4–7 Hz; Margrie and Schaefer, 2003), and delta (1–4 Hz; Schoppa and Westbrook, 2001; Urban and Sakmann, 2002) oscillations in the OB. Oscillations are thought to have a fundamental importance to OB function and have been a consistent focus in OB electrophysiology since the early work of Adrian (1942). Correlation has been shown between synchronized neuronal activity in insect antennal lobe and discrimination (Stopher et al., 1997). Studies of synchronization in MC activity have concentrated on pairs of cells from experimental recordings (Schoppa and Westbrook, 2001, 2002; Margrie and Schaefer, 2003; Ma and Lowe, 2010; Giridhar et al., 2011) and modeling (Migliore et al., 2005). It has been suggested that oscillations enhance stimulus discrimination by providing a consistent reset that prevents the accumulation of errors (Schaefer et al., 2006).

Experimental recordings of MCs have revealed a range of different firing patterns from isolated spikes to burst firing (Heyward et al., 2001; Urban and Sakmann, 2002; Margrie and Schaefer, 2003; Ma and Lowe, 2010). These firing patterns result both from intrinsic properties and external influences. MCs are known to have a wide range of channel mechanisms (reviewed in O'Connor and Jacob, 2008). The Bhalla and Bower model (Bhalla and Bower, 1993) made use of implementations for high threshold calcium, and KCa channel mechanisms along with a sub membrane calcium ion pool. Bhalla and Bower (1993) used “brute force” fitting techniques to plot the parameter space for the densities of the calcium mechanisms. Brute force fitting techniques involve mapping out the whole parameter space so the regions of best fit can be studied. Their parameters were fitted to current-clamp recordings in the presence of channel blocking agents (Mori et al., 1981; Jahr and Nicoll, 1982).

In this study we first set out to determine the PPs of MCs to be able to use them in the Migliore et al. (2005) model for paired MCs. We then aimed to extend their model to a network of 6 GJ connected MCs and determine the consequences of adding the high threshold calcium and KCa channels from the Bhalla and Bower (1993) model.

## Methods

### Electrophysiology

Sprague-Dawley rats (P21–P28) were anaesthetized with isoflurane and decapitated. Acute OB slices (300 μm) were cut in ice-cold slice solution in the horizontal plane (MICROM-HM650V, Zeiss) and incubated for 45–60 min at 34°C prior to recording. MC soma and apical dendrites were visualized by infrared differential interference contrast (IR-DIC) video microscopy and recorded from with low resistance (7–12 MΩ) glass pipette electrodes in the whole-cell patch-clamp configuration. Gigaseal cell-attached configuration was obtained first and subsequently the membrane-patch was removed to obtain a whole-cell recording. The voltage signal (filtered/sampled 6 kHz/50 kHz) was amplified with a Multiclamp 700A (Molecular devices) connected to a Macintosh computer through an ITC-18 board (Instrutech). Data acquisition and current injection was done with the Nclamp/NeuroMatics electrophysiological software (J. Rothman, www.thinkrandom.com/) built to run under the IGOR Pro scientific graphing and data analysis software (www.wavemetrics.com/). Bridge balancing and pipette capacitance compensation were done on the current injecting electrode by visual inspection on an oscilloscope of the charging of the membrane in response to −200 pA (100 Hz) square pulse injection. Only experiments with an access resistance below 40 MΩ were used. All recordings were done at physiological temperature, 33–35°C. The internal pipette solution contained (mM): 130 methanesulphonic acid, 10 Hepes, 7 KCl, 0.05 EGTA, 2 Na_2_ATP, 2 MgATP, 0.5 Na_2_GTP, 0.4% biocytin and was titrated to pH 7.2 with KOH. The slice solution was composed of (mM): 125 NaCl, 2.5 KCl, 2 CaCl_2_, 1 MgCl_2_, 25 NaHCO_3_, 1.25 NaH_2_PO_4_, 25 D-glucose and was perfused with 95%/5% 0_2_/CO_2_. The external recording solution included (mM): 105 NaCl, 12.5 KCl, 26^2^ NaHCO_3_, 1 MgCl_2_, 1 CoCl_2_, 0.001 TTX, 0.05 PicroToxin, 0.01 NBQX, 0.01 D-APV, 25 D-glucose, titrated to pH 7.4 with 95%/5% 0_2_/CO_2_. This solution was designed to block all active currents. All chemicals were from Sigma and Tocris.

Voltage responses for use in the PP fitting procedure were obtained by performing simultaneous patch-clamp injection/recording from two sites; one pipette in the soma the other in the apical dendrite. Depolarizing or hyperpolarizing current injection pulses were applied to one of the pipettes for 0.5 ms after 50 ms baseline recording. The recording was continued until 200 ms and repeated 10–20 times. The combination of depolarizing or hyperpolarizing injection current pulses and the two locations for injection or recording gives four separate groups of traces with which to constrain the parameter fits. Recordings were scaled to give superimposed baselines for the four recordings. Only transfer voltage responses (i.e., voltage recordings made from a electrode at a distance from the electrode used to inject the current pulse) were fitted while determining the PPs, thus we did not use the local voltage response derived from the current injecting electrode in order to minimize possible errors originating from pipette artifacts.

### Histology

**Figure 1:**
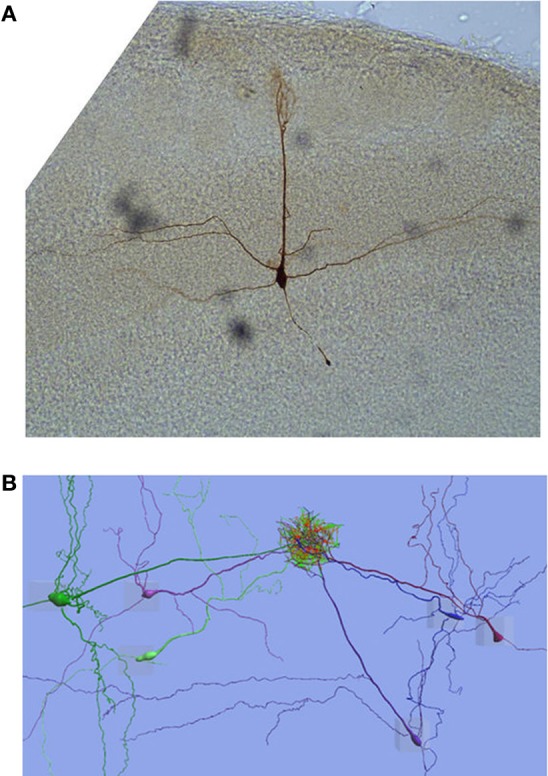
**Mitral cells. (A)** A single mitral cell from the rat olfactory bulb prepared as described in Methods (Histology). **(B)** The reconstructed MCs in the 6 cell apical dendrite tuft gap-junction network model come from different animals so, to enmesh the apical dendrite tufts, the apical dendrites end up in many different orientations when they would naturally be almost parallel. Gap-junction links are represented by lines that transition from red at one end of the connection to green at the other end of the connection.

The structure of each MC recorded from was obtained by filling the cell with biocytin through the whole-cell pipette and subsequently processing the slices histologically (see Figure 1A). Immediately after ending the recording, the patch pipettes were carefully withdrawn from the dendrite/soma and the slice was fixed for a minimum of 24 h in a 4% paraformaldehyde phosphate buffered solution (PBS) at 4°C. The membranes were permeabilized with pre-cooled 100% methanol at −20°C for 10 min. Endogenous peroxidase activity was blocked with 1% H_2_O_2_/10% methanol and the signal was amplified by incubation for 18–24 h in the ABC kit solution (Vectastain). The slices were washed thoroughly with PBS between each step. After a DAB (3,3-diaminobenzidine hydrochloride) amplification (1 mg/ml, 15 min at room temperature) the stain was developed by adding H_2_O_2_. The progress of the staining was followed under the microscope to ensure a dark brown, not completely black, coloration of the dendrites, which we found was optimal for digital tracing of the full morphology. The slices were mounted under coverslips with Mowiol 4-88 medium (Calbiochem).

### Morphological reconstruction

Neurolucida (MicroBrightField, Colchester, VT, USA) reconstructions were produced using a light microscope (BX-61, Olympus) with 100× oil immersion objective. The axon and dendrites are represented by diameters located by 3D coordinates and the soma by a 2D outline.

The inter pipette distances were estimated from DIC images obtained during experimentation.

The amount of shrinkage produced by the histological methodology used for fixing the slice has not been estimated. Therefore, as a worst case scenario a modified version of the reconstruction was produced for comparison in which 50% shrinkage was simulated by doubling the z-plane within Neurolucida.

### Compartmental modeling

Simulations were carried out using the “Neuron” simulator (Hines and Carnevale, 1997, 2000). Neurolucida reconstructions were converted to “Neuron” parameter files using Neurons' built in Import3d tool. The spatial segmentation was handled by the d-lambda rule setting a value for d-lambda of 0.1 (Hines and Carnevale, 2001).

Later compartmental models in Neuron where mostly created with the help of neuroConstruct (Gleeson et al., 2007) 3D. This tool automates some of the aspects of model specification which allows a degree of randomization in synapse location. Its 3D visualization allows rotation of cells for inspection of their morphology.

### Fitting passive parameters

In compartmental models each compartment is modeled as an equivalent circuit which includes values for *C*_*m*_, *R*_*m*_, and up and downstream *R*_*i*_. Normally this equivalent circuit would also include values for active conductances, but these are not used for a passive model. So a differential equation for each compartment can be given as:

$$C_{m}dV_{m}/dt=(E_{m}-V_{m})/R_{m}+({V^{\prime }}_{m}-V_{m})/{R^{\prime }}_{t}+({V^{\prime \prime }}_{m}-V_{m})/R_{i}+I_{\text{inject}}$$

for a fully labeled equivalent circuit see Bower and Beeman (1998), the apostrophies are used to indicate up and down stream membrane voltages or axial resistance.

We have chosen to fit *C*_*m*_, *R*_*m*_, and *R*_*i*_. As we are using whole-cell patch-clamp recordings we are assuming that there is no need for somatic shunt resistance *R*_*sh*_ (Clements and Redman, 1989; Major et al., 1994). Also we have made no attempt to fix *R*_*i*_ to assumed values (Clements and Redman, 1989; Major et al., 1994).

These passive values were directly fitted to current injection pulse transients within the 3D rat MC morphological reconstructions. To enable the concurrent fitting to four separate recordings (depolarizing or hyperpolarizing current injection into the soma and recorded in the apical dendrite or the other way round as detailed under electrophysiology) two Neuron *point process managers* were used in the simulation to mimic the input conditions at the soma and the nearest point to the distance between pipettes that the *point process manager* would allow (Table 3). The PPs generated from the fits, like the experimental recordings, produce simulations for current injection into the soma and recorded in the apical dendrite that can be superimposed on a recording made in the soma from similar injections in same location in the apical dendrite. Under passive conditions these recordings superimpose, a phenomenon known as reciprocity. A further confirmation of passivity is linear scaling. To test this, simulations were carried out with current injections of 0.5 and 1.0 nA to determine whether the 0.5 nA current response superimposes on that in response to the 1.0 nA injection.

No allowance has been made to add increased surface area to the morphology to compensate for the surface area of spines as there is no evidence for the presence of spines in MCs (Price and Powell, 1970; Nagata, 1989).

### Active properties

The Migliore model (Migliore et al., 2005) Na, K_dr_, and K_A_ *Neuron* channel mechanism scripts were converted to XML versions (ChannelML). This is to enable the use of combinations of mechanisms from Genesis channel scripts, particularly those from the Bhalla and Bower (1993) model, as the model develops. It is possible using neuroConstruct to produce code in either the Neuron or Genesis script languages (http://www.neuroml.org/). To validate these conversions, as being true copies that impart the exactly the same properties as the originals, direct comparisons of the original Neuron and Genesis versions of the mechanisms and XML versions were made in neuroConstruct comparing voltage response and internal variables in a single segment cell (Gleeson et al., 2007).

The KCa channel proved problematic. It was originally created using an obsolete Genesis object called a “vdep” channel. The “vdep” channel was incompatible with integrator routines that speed up the running of calculations during simulations so was replaced by tabchannel and tab2Dchannel mechanisms (these are Genesis objects that calculate Hodgkin–Huxley type channel equations with 1 and 2 dimensional tables, respectively). A close fit could only be achieved by a ChannelML implementation if a two gate approach was taken. The justification for this is that in the original Genesis implementation the voltage-dependent part of the equation is handled as a separate differential equation to fill a table as if it was a separate gate but with just an α value. However, in channelML implementations both gates must have a β value. It was found that the curve could be fitted using values for m β and z β.

### Network connections and firing synchronization test

Network connections were created using GJs between MC apical tufts. The morphologically reconstructed cells were taken from different animals and therefore the apical dendrites are not orientated in the same way, as would be the case if they had all come from the same glomerulus in the same animal. However, using neuroConstruct to spatially re-position the MCs it was possible to partially enmesh the apical tufts (Figure 1B).

The GJs (parameters from Migliore et al., 2005) were incorporated into the apical tuft segments with 100 network connections established between pairs of MCs using a networking facility built into neuroConstruct. Immunocytochemical studies of OB glomeruli have succeeded in detecting GJs between MC intraglomerular dendrites in mice (Kosaka and Kosaka, 2004; Christie et al., 2005; Rash et al., 2005) but as yet these studies are limited qualitative studies. For this reason networks with 1, 10, and 100 GJs with coupling ratios (ratio based on voltage deflection recorded test cell: stimulated cell, see below) of around 0.04 were compared. Only networks with 100 GJs produced synchronization of firing. This is likely to be due to the conductance being distributed evenly across the ADT thereby more closely mimicking the input. Hundred GJs per pair gives a total of 1600 GJs in a six cell network and GJ conductance was adjusted to match experimentally observed coupling ratios of between 0.01 and 0.08 (Schoppa and Westbrook, 2002). To measure coupling ratio a −0.3 nA hyperpolarizing current injection with a 50 ms delay and 150 ms duration was injected into the soma of the pre-MC and the somatic voltage displacement for the pre- and post-GJ MCs were compared.

### Synchronization test

Testing for the synchronization of firing between MCs by GJs was carried out using the Migliore protocol (Migliore et al., 2005). Current injections were made at 20 randomly placed locations in the apical tufts of two connected MCs. This figure of 20 input sites comes from the design feature of the Migliore model called a Rallbranch (referring to the number of “virtual” copies of a section and its subtree, including all synapses, that connect to the parent) that allows multiple identical branches to have the same settings by setting up a single unit. This was used to setup the ADT and a single input site therefore gives 20 inputs. We have tried a single site rather than 20 and 20 gives better synchrony. The actual number of inputs is unknown and likely to be a large number so we took the pragmatic approach and used 20 to make our results comparable to the Migliore models.

These continuous depolarizing current injections were just above threshold and produced tonic firing in the MCs. Using 20 locations for current injection allowed lower current to be applied to the individual locations thereby preventing individual GJs' maximum current transmission from limiting the total transmission between the cells. This mimics to a small degree the large number of inputs from olfactory receptor neurons but, of course, the order of magnitude of individual inputs is very much greater. Upwards of 10,000 olfactory receptor neurons converge on the ADTs of around 80 MCs (Alison and Warwick, 1949). However, the densely packed and complex nature of the glomerulus (see O'Connor and Jacob, 2008, for discussion of juxta- and intra-glomerular cells) has prevented an estimation of the number of synapses from an individual olfactory receptor neuron on to ADTs of MCs. With 20 inputs the current was low enough on individual inputs to allow sufficient flow across the GJs to give coupling ratios equivalent to those found by experimental measurement (between 0.01 and 0.08; Schoppa and Westbrook, 2002).

The current injection was applied to both MCs, but the start of the current injection was delayed by 10 ms (Migliore et al., 2005 protocol) for one of the MCs. If the MCs were not connected, or only weakly connected by GJs, this offset in the start of current injection would produce desynchronized firing, with the same 10 ms spike time difference between firing in the two cells.

Three different methods were used to assess the degree of synchrony: cross correlation, principal component analysis (PCA), and spike time differences:

1. Cross correlation assesses pairs of spike trains that have been converted from the recorded data into binary vectors. The vectors consist of a series of time bins in which a zero is found if there is no spike event within the time interval, and one if there is a spike event in the time interval. In the cross correlation, lag is used to denote the shift along the time axis (ms) needed for a spike in one vector to line up with a spike in the other vector. The y-axis in the cross correlogram is the number of examples of spike pairs that line up for a particular unit of lag.
2. PCA is a statistical method that reduces the dimensions in a data set so as to be able to apportion variance between components as eigenvalues (EV). It finds sequentially a specified number of components in which most of the variance lies. Adding the EV of the components together gives a number equal to the total number of components, which is 100% of the variance. Here we use paired comparison which allows two components of variance. Variance is at its lowest when the majority of the variance is with the first component. So for a paired comparison of two recordings, perfect synchrony would be when the first component has an EV of 2. PCA was performed on pairs of complete 300 ms *V*_*m*_ waveforms and in some cases EV approach 2 indicating high degree of synchronization.
3. Spike time difference comparisons were made by finding the times of the peak voltage and finding the mean of the differences for all the peaks in a 300 ms recording. Unlike the PCA method it does not compare the full time course of the recordings.

## Results

**Figure 2:**
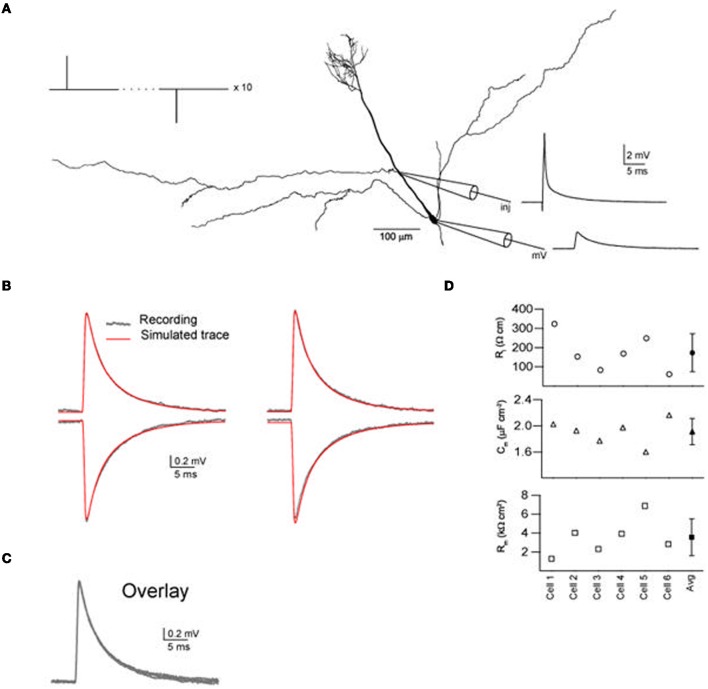
**Direct fitting of passive parameters for rat olfactory bulb mitral cells using short current clamp injections and Neurolucida reconstructions in a dual recording site protocol. (A)** One of the mitral cells (cell 2) showing the positions of the input current injection (cycling between hyperpolarizing and depolarising current injections, see Methods) patch-clamp electrodes and experimental recordings. **(B)** Experimental recordings from the distal and proximal apical dendrite recording sites for depolarizing and hyperpolarizing current injections at the alternate electrode locations. Superimposed are the fitted curves produced with the best fit parameters found by the PRAXIS algorithm using the Neuron simulator, showing that the model gives a good fit for the experimental recordings. **(C)** All four experimental recordings in **(B)** superimposed as a test to show that the blocking conditions used had resulted in the cell exhibiting passive behavior. **(D)** The fitted passive parameters *R*_*i*_, *C*_*m*_, and *R*_*m*_ for each of the six cells are all of the same order of magnitude (for actual values and τ_*m*_ values see Table 1).

Six MCs were selected for parameter fitting based on the quality of electrophysiological recordings and the proximity of resting potential to −60 mV. Detailed reconstructions using Neurolucida were made for the six cells (the range of morphological dimensions is summarized in Table 1). Direct fitting to dual current clamp pulse recordings (Figure 2A) of PPs [intracellular restivity (*R*_*i*_), specific membrane capacitance (*C*_*m*_), and the specific membrane resistance (*R*_*m*_)] were made using the PRAXIS fitting algorithm in Neuron (see “Methods”). The parameter values were always determined by fitting to experimental recordings using digital reconstructions from the same cell.

**Table 1:** **Morphological dimensions, passive parameters, and membrane time constant (τ_*m*_) of the six olfactory bulb mitral cells used in the study**.

	**Cell 1**	**Cell 2**	**Cell 3**	**Cell 4**	**Cell 5**	**Cell 6**
Inter-electrode distance citation (μm)	116	125	100	133	206	137
Sections	53	117	131	49	71	37
Segments	839	2417	1740	1072	2018	1161
Total surface area (μm^2^)	12,703	15,499	13,823	10,493	25,427	14,754
Tuft surface area (μm^2^)	1934	2858	4898	1560	888	1145
Soma surface area (μm^2^)	1309	799	887	749	2331	1019
Diameter of Proximal end of apical dendrite trunk by the soma (μm)	3.00	3.22	2.48	1.81	2.55	1.81
Diameter of apical dendrite trunk at electrode location (μm)	1.44	1.30	1.11	1.07	1.30	1.30
*R*_*i*_ (Ω cm)	322.92	153.23	83.05	168.94	248.95	62.27
*C*_*m*_ (μF cm^−2^)	2.03	1.93	1.77	1.97	1.60	2.16
*R*_*m*_ (Ω cm^2^)	1275.8	4099.9	2301.3	3914.7	6867.1	2823.1
τ_*m*_ (msec)	2.58	7.90	4.08	7.73	10.98	6.11

Prior to the recordings, synaptic, and ionic activity were blocked by adding specific blockers to the bath solution (see “Methods”). Also we tested whether the cells expressed the hyperpolarization-activated cation current (*I*_*h*_) by injecting hyperpolarizing steady state pulses (1 s) to 20–25 mV below the resting potential. None of the six cells chosen for parameter fitting exihibited the rebound sag characteristic of the *I*_*h*_ current. Under these conditions the membrane acted in a highly linear and reciprocal manner, where the transfer voltage responses of the dendrite and the soma from current injection in either location and at different amplitudes showed almost perfect superimosition when scaled by 1/current (Figure 2C), implying that the membrane was passive. We achieved accurate fits to the voltage responses (Figure 2B) obtaining PP values for all six cells which where in the same order of magnitude (Figure 2D). In comparison with published figures for other brain regions *C*_*m*_ is high and *R*_*m*_ is low (Table 2). The fitting of PPs to steady state recordings with current injections of 50 ms gave values in the same range (*R*_*i*_, 98–251 Ω cm; *C*_*m*_, 1.9–2.6 μF cm^−2^; *R*_*m*_, 1.4–7.9 k Ω cm^2^).

**Table 2:** **Comparison of mitral cell passive parameters with those of other neurons**.

**Reference**	**Cell type**	**Sample size**	***R*_*i*_ range Ω cm**	***C*_*m*_ range μF cm^−2^**	***R*_*m*_ range KΩ cm^2^**
Our results	Rat mitral cell	*n* = 6	62–323	1.6–2.1	1.3–6.9
Migliore et al., 2005	Mitral cell	−	150	1.8	12
Major et al., 1994	Rat CA3 pyramidal neurons	*n* = 4	170–340	0.7–0.8	120–200
Thurbon et al., 1998	Rat ventral horn neurones	*n* = 4	87 ± 22	2.4 ± 0.5	5.3 ± 0.9
Stuart and Spruston, 1998	Rat neocortical pyramidal neurons	*n* = 3	70–100	1.1–1.5	35-5 (non-uniform)
Roth and Häusser, 2001	Rat cerebellar purkinje cells	*n* = 4	115 ± 20	0.77 ± 0.17	122 ± 18
Golding et al., 2005	Rat CA1 pyramidal neurons	*n* = 3	139–261	1.02–2.02	10.2–125.6
Perreault and Raastad, 2006	Rat lateral geniculate nucleus Thalmocortical cells	*n* = 15	22–424	median 1.2	20–53
Perreault and Raastad, 2006	Rat lateral geniculate nucleus interneurons	*n* = 3	median 113	median 1.2	83–124

To estimate the confidence limits imposed on the results by various sources of error we carried out a detailed assessment of potential sources that will be presented over the next few paragraphs. In the discussion section some further sources of error will be covered that we felt had been successfully dealt with by other authors.

To be sure that the PRAXIS fitting algorithm, combined with our cell reconstructions, are capable of consistent and accurate fitting, even at extreme parameter combination, we used a series of target parameters to produce simulations using the individual MC reconstructions. In these simulations we mimicked the experimental conditions by using injected current pulses for depolarizing and hyperpolarizing current injections of 0.45 nA at the apical dendrite while recording at the soma or visa-versa. The groups of simulated recordings were then used to test the PRAXIS PP fitting and the resulting fits were compared with the target figures. All six reconstructions produced perfect fits (typical error values for least square comparisons between target curve and simulation were 2.5 × 10^−9^) to the curves and a precise match of the target parameters, showing that there is no error stemming directly from the algorithm or artifacts induced by the use of the cell reconstructions. This was true for target parameter values chosen to cover *R*_*m*_ and *C*_*m*_ values found in the other studies.

In the MC with its long, sparsely branching, apical dendrites, it is especially difficult accurately to establish the location of the pipette. There are two sources of error related to the pipette location, an experimental error and a modeling error. The experimental error relates to making inter-pipette distance estimates. Pipette locations were identified by estimating inter-pipette distances on IR-DIC images. One pipette was always located in the soma, the other pipette was located on the apical dendrite at a distance measured from the soma. We therefore think that a reasonable error of margin to attribute to the DIC image estimate of inter-pipette distances is ± 10%. The modeling error relates to the spatial segmentation of the model. In “Neuron”, to minimize the computational load, the membrane current and potential are only calculated at one or more discrete positions (“nodes”) that are equally spaced along the interior of a section. In the simulation, “Point Process Managers” that handle current injection can only be located at these nodes. To minimize this error we adjusted the number of nodes in the relevant section of the model's apical dendrite to locate the current injection and recording as close as possible (see Table 3 for actual figures) to the DIC estimate.

**Table 3:** **The difference between inter-electrode distances estimated from DIC images and the positions on the simulations**.

	**Cell 1**	**Cell 2**	**Cell 3**	**Cell 4**	**Cell 5**	**Cell 6**
Experimental inter-electrode distance (μm)	116	125	100	133	206	137
Simulation inter-electrode distance (μm)	114.43	128.89	100	139	205	137.11
Percentage difference	−1.35	3.11	0	4.97	−0.24	0.08
Percentage electrode offset (minus)	−10.26	−10.63	−10.36	−9.88	−10.53	−9.69
Percentage electrode offset (plus)	10.53	9.65	10.36	10.28	10.53	9.75

The simulation electrode positions are dictated by the positions of nodes which result from dividing the section into a specified number of segments. The electrode offsets were produced to estimate electrode offset error in passive parameter fitting. The aim was a 10% electrode offset and the actual values are what could be achieved within the node restrictions.

**Figure 3:**
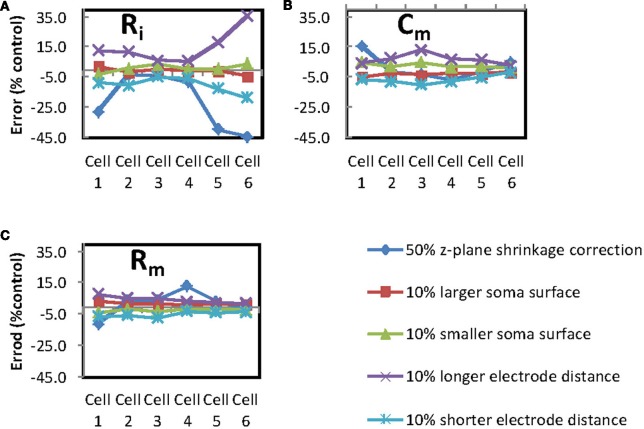
**Determination of possible sources of error.** Percentage error was calculated for each of the passive parameters for comparisons between a control of the standard conditions for fitting each of the passive parameters and modified conditions as follows: **(A)** Error from *R*_*i*_, **(B)** error from *C*_*m*_ and **(C)** error from *R*_*m*_, error generated from 50% z-plane shrinkage correction (dark blue line), 10% larger soma surface area (red line), 10% smaller soma surface area (green line), 10% greater inter-electrode distance (purple line), 10% shorter inter-electrode distance (light blue line).

The electrode distance data points on Figure 3 (purple and light blue lines) represent the effect on the three PPs from a ±10% offset in pipette location that we have assigned as the error margin for the DIC inter-pipette distance estimates. The error bar values were produced by running simulations with the fitted parameter values and the DIC estimate pipette locations. These simulated curves were then fitted with both a + and −10% offset in pipette location [within the limits of what was possible with section segmentation (see Table 3 for actual percentage electrode offsets)].

### Shrinkage correction

The cell reconstructions were made on fixed preparations for which there will be a degree of shrinkage in comparison to the living slice used for experimentation. In the absence of good estimates of the degree of shrinkage produced by the fixation methodology used, we looked at the effect of 50% shrinkage. A doubling of the z-plane values in the reconstruction was used to simulate 50% shrinkage (shrinkage correction data points are dark blue lines on Figure 3). It is unlikely that shrinkage was as severe as this but a large figure gives us a chance to observe the impact of shrinkage with a high safety margin.

### Soma surface area

Reconstruction of the soma in Neurolucida is restricted to putting a 2D outline round a single (shifting) plane of focus. When this outline is imported into Neuron using the Import3d tool, a series of diameters perpendicular to an axis are used to create a 3D interpretation of the 2D outline. This approach produces a soma comprising about 20 segments that the simulator can handle, but cannot be claimed to be an accurate estimation of the soma surface area. To assess the importance of soma surface area in fitting accuracy, we increased and decreased all the diameters in segments forming the soma polygon by 10% and performed fits with the new dimensions (soma surface area data points are the red and green lines on Figure 3).

The error plots in Figure 3 show that the intracellular resisitivity (first chart) is the PP which, out of the three, was most sensitive to experimental and simulation inaccuracies. Logically the *R*_*i*_ values in particular are very prone to be influenced by changes in the inter-electrode distance error and shrinkage correction.

### General comments on sources of error

The noticeably larger errors are found to affect *R*_*i*_, for cells 1, 5, and 6. There does not appear to be obvious differences between the morphology of cells 1, 5, and 6 and cells 2, 3, and 4 that would explain the first group having larger *R*_*i*_ for shrinkage correction. Table 1 shows some morphology measurements; see, in particular, the inter-electrode distance and apical dendrite diameters at the proximal end and at the electrode positions as these are the measurements most likely to affect fitting errors.

**Figure 4:**
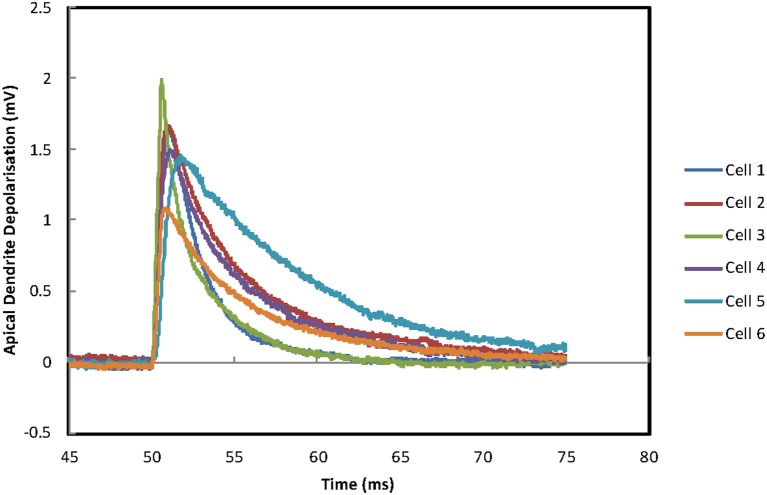
**Whole-cell patch-clamp data.** Experimental recordings in the soma from depolarizing current injections into the distal apical dendrite. This is one of the four conditions that were used for fitting the passive parameters (see legend to Figure 2).

It is also difficult to spot any differences in the experimental recording used for fitting (Figure 4) that might explain the observed increased magnitude of *R*_*i*_ error for Cells 1, 5, and 6 with shrinkage correction. Cell 5 exhibits a slower depolarization time course than the other recordings. But this is not the case for cells 1 and 6. We can therefore only conclude that for *R*_*i*_ fitting with shrinkage correction there is a complex relationship that will increase the observed error for a subset of cells.

### Paired mitral cell model

**Figure 5:**
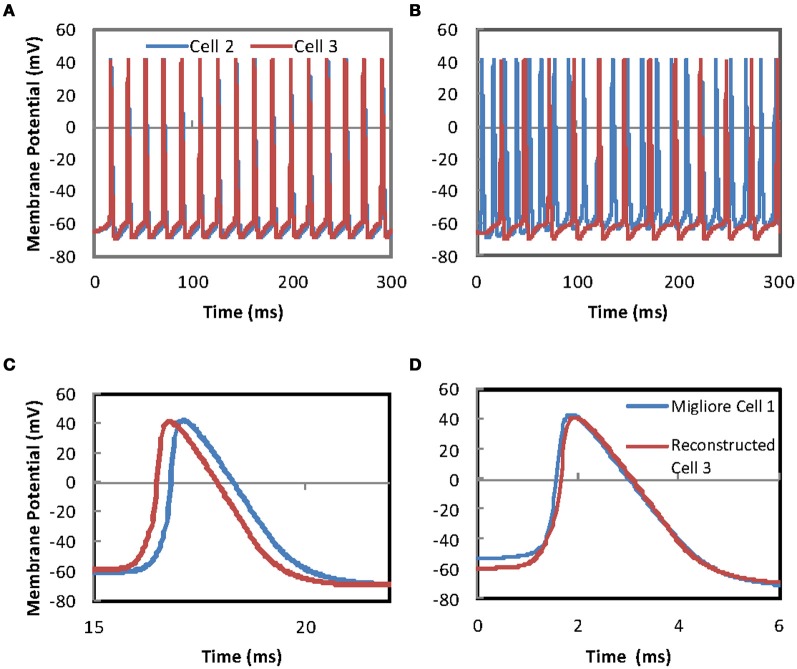
**Firing in a pair of MCs is synchronized by gap-junction connections between the apical dendrite tufts of the two cells. (A)** Simulation of somatic membrane potential in two reconstructed MCs with fitted passive parameters and Na, K_dr_, and K_A_ channel mechanisms derived from Migliore et al. (2005). The MCs are connected by 100 gap-junctions between the apical tufts of the pair of cells. The MC are given current injections such that if the gap-junction connections were not present the two cells would produce asynchronous firing (see “Methods”) **(B)** Asynchronous firing in a pair of MCs with no gap-junction connections. Simulation of somatic membrane potential in two reconstructed MCs with fitted passive parameters and Na, K_dr_, and K_A_ channel mechanisms derived from Migliore et al. (2005). Cell 2 is given a constant current injection to 20 locations to give tonic firing. Cell 3 receives the same current injection to 20 locations but the start is delayed by 10 ms. Unlike the simulation for Figure 5A there are no gap-junctions in this set-up. **(C)** Synchronization of firing is immediate, however, the sub-threshold depolarizing phases do not synchronize due to the differences in passive parameter of the two cells shows an enlarged time scale version of **(A)** to allow the size of the offset between the two to be viewed. **(D)** Action potential time course comparisons show that MC morphology has little effect on action potential duration. Superimposed simulation traces comparing the action potential timing for MCs with the canonical morphology used in Migliore et al. (2005) and our reconstructed morphology. The “Reconstructed Cell 3” is the same simulation trace depicted as Cell 3 in Figure 5A. The faster time course for the rising phase of the action potential for the Migliore cell is morphology related, where rising phase timing is proportional to distance between current injection and the soma.

The passive cell models were now used to construct a gap junction connected network. Initially just two MCs were included in the simulations giving a coupling ratio (see “Methods”) of ~0.044 (Figure 5A). This coupling ratio is somewhat higher than the coupling ratio achieved in the Migliore model (Migliore et al., 2005; measured from model forfig6-modeldb.hoc as published at http://senselab.med.yale.edu/modeldb/ShowModel.asp?model=43039 ~0.031). Synchronization of spiking in the 2 cell simulation was immediate but the difference in PPs means that the sub-threshold depolarization remains asynchronous. This is not the case in the Migliore model (Migliore et al., 2005) where complete synchronization occurs because the PPs of the two cells are identical. Notwithstanding the sub-threshold asynchrony, comparison between a simulation with (Figure 5A) and without (Figure 5B) the GJs reveals that the degree of synchronization mediated by the GJ is substantial. Experimental assessment of correlated firing between pairs of MCs (Schoppa and Westbrook, 2002) used the ratio of probability of spikes from two cells in a cross correlogram that fall within 10 ms of each other versus between 30 and 40 ms of each other. This type of probabilistic criteria is suited to experimental work on neurons but cannot be applied to simulations that produce the same result every time while parameters remain the same. However, for the sake of comparison it can be noted that 20 ms bins were used to produce the cross correlograms in experimental studies on neurons (Schoppa and Westbrook, 2002) while 1 ms bins where needed to separate the spike trains in Figure 5A. From the cross correlogram the mean absolute lag (see “Methods”) for the pair of spike trains in Figure 5A was 0.44 ± 0.75 ms which is an order of magnitude better synchrony than the *in vitro* studies. For comparison the cross correlogram performed on the pair of MCs minus the GJs in Figure 5B has a mean absolute lag of 4.29 ± 0.83 ms.

At ~ 2–3 ms in duration the APs modeled have a slower time course than measured experimental durations of ~ 0.8–1 ms (e.g., Chen et al., 1997; Christie and Westbrook, 2003). This extended duration is a function of the gate kinetic equations for the Na, K_dr_, and KA ion channels in the Migliore et al. (2005) model. In Figure 5D a comparison is made by superimposing simulations using the reconstructed morphology and the simple morphology used by Migliore et al. (2005). The distance between the apical tuft and the soma is shorter in the Migliore et al. (2005) morphology than the reconstructed morphology. This produces a faster time course in the rising phase of the AP for the Migliore et al. (2005) morphology. However, the overall duration of APs is unaffected by morphology (Figure 5D). It would be desirable in the future to have more experimental measurements of channel gating kinetics to be able to have more realistic AP time course properties.

### Synchronization in a 6 cell model

**Figure 6:**
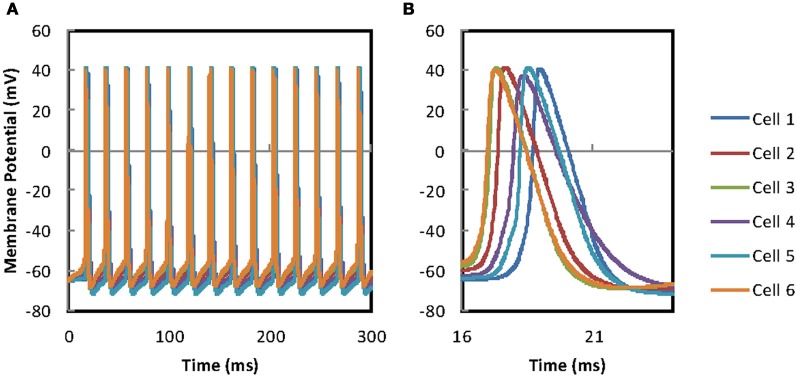
**MC network synchronization. (A)** The current injection evoked APs in the original pair evoke APs across the population of gap-junction connected MCs. Four more gap-junction coupled MCs have been added to the pair of MCs in Figure 5A. The original pair of mitral cells receive the same input as described in Figure 5A. Firing of all cells is tightly grouped but full synchronization is not achieved. **(B)** An enlarged time scale version of **(A)** to allow the size of the offset between the two traces and the timing of the AP to be viewed.

For the 6 cell model, four more MCs are added to the unchanged 2 cell model. GJ connections are added to all pairs (see “Methods”), but current injections are not made to the additional cells. For the same range of GJ conductances coupling ratios are now lower at ~0.025 (the increase in coupling ratio with increasing GJ conductance ceases at ~0.03). This reduction of coupling ratio is produced by the extra four cells in the circuit absorbing some of the current by acting as a current sink. For these coupling ratios all the connected cells without direct current injection produce GJ driven action potentials (Figure 6A). Spiking is fairly tightly grouped (range of spike times ~2 ms; Figure 6B) but falls short of the synchrony in the pair of cells (spike time difference < 0.5 ms; Figure 5C). The differences in the sub-threshold depolarizing phase reflect the range of PPs across the 6 MCs.

Cross correlation and measurement of spike-time differences (see “Methods”) were used to investigate in more detail the effect of PPs on network synchrony. Using cross correlation it was found that MCs in the network, when fitted with their own PPs (their fitted values for *R*_*i*_, *C*_*m*_, and *R*_*m*_), generated poorer synchrony across the network than when MCs shared identical PPs. Spike time differences for MC pairs dropped from above 1 ms for MCs with different PPs to below 0.5 ms for pairs with the same PPs. This difference is confirmed with PCA where MCs with their own (different) PPs have a significantly different mean EV of 1.66 SD ± 0.25 while MCs with identical PPs have mean EV of 1.88 SD ± 0.11 (*P* = 0.004) (perfect synchrony, EV = 2).

### Effects of individual passive parameters

**Figure 7:**
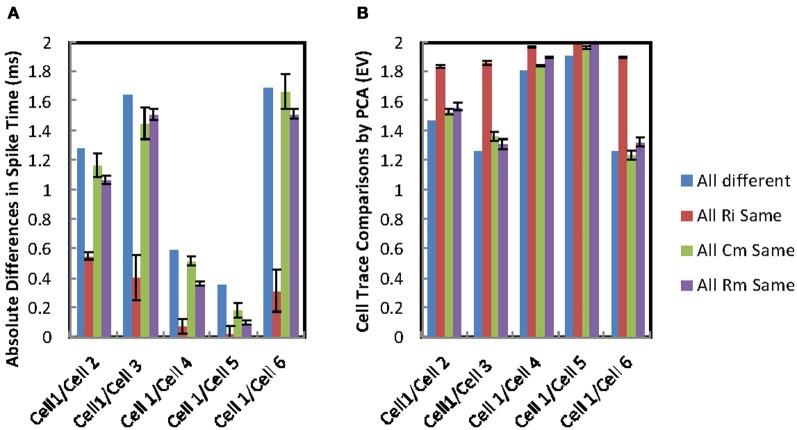
**Synchrony in gap-junction coupled MCs. (A)** Spike time differences for comparisons of somatic membrane voltage recordings for cell 1 and the other cells in simulations from a 6 MC apical dendrite tuft gap-junction connected network model. The groups compare simulation set-ups in which all MCs have their own different passive parameters with simulation set-ups in which one of the passive parameters is set to identical values for all of the cells. The differences where all passive parameters are different are the means of all peaks for a single simulation. While those for one identical passive parameter is the means of all peaks from six simulations each with a different setting for the fixed passive parameter. **(B)** Principal component analysis (PCA) analysis of synchrony demonstrated that cells with identical *R*_*i*_ exhibit better synchrony (red columns EV closer to 2). Eigenvalue differences for comparisons of somatic membrane voltage recordings for cell 1 and the other cells in simulations from a 6 MC apical dendrite tuft gap-junction connected network model. The groups compare simulation set-ups in which all MCs have their own different passive parameters with simulation set-ups in which one of the passive parameters is set to identical values for all of the cells. The differences where all passive parameters are different are PCA comparisons of 300 ms simulations of somatic membrane potential. While those for one identical passive parameter are the means of PCA comparisons of 300 ms simulations of somatic membrane potential from six simulations each with a different setting for the fixed passive parameter.

The influence of individual PPs were explored by varying one of the PPs while the remaining PPs were kept at the MC's own fitted values. The 6 cell model introduced in the previous section was systematically changed so that in turn each one of the PPs is selected and set to a fixed value on all the cells while the other two PPs are allowed to vary. All of the fitted PP values for the individual MCs were used in turn as the value the cells are fixed to. The resulting spike time differences and PCA for individual pairs were compared. For the 6 cells there are 15 possible pair comparisons, but for clarity the figures show only the comparisons between MC 1 and the other cells in the model. MCs with identical *R*_*i*_ achieve a drop in spike separation for pairs to approximately 0.5 ms or less while those with identical *C*_*m*_ or *R*_*m*_ only rarely are better than 1 ms (Figure 7A). Spike time differences between MC 1 and MCs 4 and 5 when all PPs are different are already small (see Figure 6B), but setting identical *R*_*i*_ values for the MCs still gives a further improvement of synchrony (Figure 7A). The same trends are observed for PCA analysis, mean EVs are: identical *R*_*i*_, 1.91 SD ± 0.06 (Ω cm); identical *C*_*m*_, 1.58 SD ± 0.28 (μF cm^−2^); identical *R*_*m*_ 1.62 SD ± 0.29 (Ω cm^2^; Figure 7B).

### Calcium channels and burst firing mitral cells

**Figure 8:**
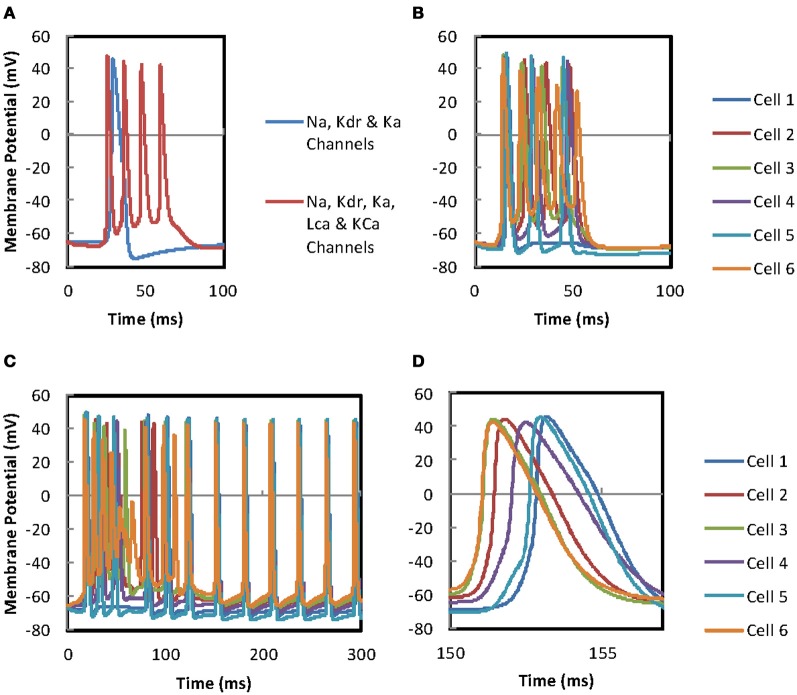
**(A)** Comparison of firing in mitral cells with and without L-type calcium and KCa ion channels. The stimulation of two identical mitral cells with current injection (0.07 nA) to the apical dendrites. Both MCs contain Na, K_dr_, and KA channels but, the cell represented by the red trace has, additionally, L-type calcium channels, KCa channels, and a calcium-pool (see text for further details). These additional mechanisms enable the cell to produce burst firing. **(B)** Burst firing in the gap-junction connected mitral cell network. Simulation of the 6 cell MC network with two sets of current injections to cells 2 and 3, 10 ms apart (see text for further details). The gap-junction coupling ratio has been adjusted ~0.025 but the cells do not synchronize. **(C)** The effect of continuous current injection to the mitral cell network. Simulation of the 6 cell MC network with a continuous current injections (0.06 nA) to cells 2 and 3, 10 ms apart (see text for further details). The gap-junction coupling ratio has been adjusted ~0.025 but the cells do not synchronize. Firing of the cells is asynchronous for the first 120 ms. However, after the burst firing phase is completed the firing of all the cells becomes synchronous. **(D)** An enlarged time scale of **(B)** allowing the size of the offset between the traces to be viewed.

The addition of the Bhalla and Bower (1993) calcium channel scripts to our single MC models that contain the Migliore et al. (2005) Na, K_dr_, K_A_ channel scripts, changes the response of MCs from a single action potential evoked by a short current injection to a burst of action potentials (Figure 8A). The high threshold calcium channel maximum activation occurs at the point of peak depolarization of the action potential and extends into the falling phase. This activation produces an influx of calcium that depolarizes the cell sufficiently to evoke further action potentials when the steady state activation of the Kdr channels approach zero. As the Ca^2+^ concentration increases the activation of the KCa channels also increases until a point is reached where the cell becomes sufficiently hyperpolarized to reduce the activation of the high threshold calcium channels. When activation of the high threshold calcium channels decreases the decay of Ca^2+^ concentration produced by a fixed decay constant in the simulation, or calcium pumps in a living cell, starts to be stronger than the influx and a point is reached where the burst firing is quenched.

### Gap-junction network and synchronization

When six cells in a GJ connected network using the Bhalla and Bower (1993) calcium channels are stimulated, the first spike for each MC is relatively tightly grouped with the firing of the other cells in the network, but inter-cell grouping breaks down in the following spikes (Figure 8B). In this simulation cell 2 received a 0.05 ms current injection of 0.5 nA after a delay of 10 ms to 20 locations in its apical dendrite to give burst firing. A second 0.5 nA current injection to 20 locations was delivered with a delay of 20 ms to the apical dendritic tuft of cell 3. The 10 ms delay between the two current injections to the two separate cells would produce asynchronous firing in unconnected cells. We use it here to test the ability of the 100 GJs between the apical tufts of the cells to synchronize firing in a small network (fewer than this did not allow synchronized firing). The coupling ratio has been adjusted to ~0.025, a level at which GJ driven firing is observed in some, but not consistently in all cells. Figure 8B demonstrates that burst firing is not synchronous between cells. This is in contrast to simulations in which calcium mechanisms are not present, where GJ mediated synchronization is more efficient (Migliore et al., 2005). The burst firing characteristics of the individual cells appear to overcome the synchronizing effect of the GJs. Also, it is not obvious what effect the second current injection into cell 3 at *t* = 20 ms is having on desynchronizing the network firing. Omitting the second current injection has no effect on firing. If the first current injection is omitted the response is shifted along the time axis reflecting the later current injection, but the waveforms remain the same. Changing the timing of the second current injection from to 17, 15, or 12 ms also produces identical waveforms apart from in the case of the 12 ms delay. In this case all the APs in the waveforms are shifted to about 0.6 ms earlier but the overall pattern remains the same. This suggests that the two current injections are integrating allowing threshold potential be achieved 0.6 ms earlier. It therefore appears that once a MC network incorporating the Bhalla and Bower (1993) calcium mechanisms receives a supra-threshold stimulus the waveform of its response is resistant to the influence of further stimuli be they direct or via GJs.

The injection of a continuous current of 0.06 nA to 20 locations in the apical tufts of cells 2 and 3 with a 10 ms delay demonstrates that the burst firing has a desynchronizing effect which lasts for around 120 ms (Figures 8C,D). However, after 120 ms all the cells settle down to a rhythmic firing pattern for which the action potentials of each of the cells are tightly grouped. Further tests with continuous current injections to a single cell (rather than two cells) in the network produced similar patterns of asynchronous burst firing followed by synchronous repetitive firing when either MC 2 or MC 3 received the current injection. The current injected needs to be doubled from 0.06 nA to 0.12 nA to evoke action potentials. The overall timing varies depending on the cell receiving the current injection and the delay before the current injection.

**Figure 9:**
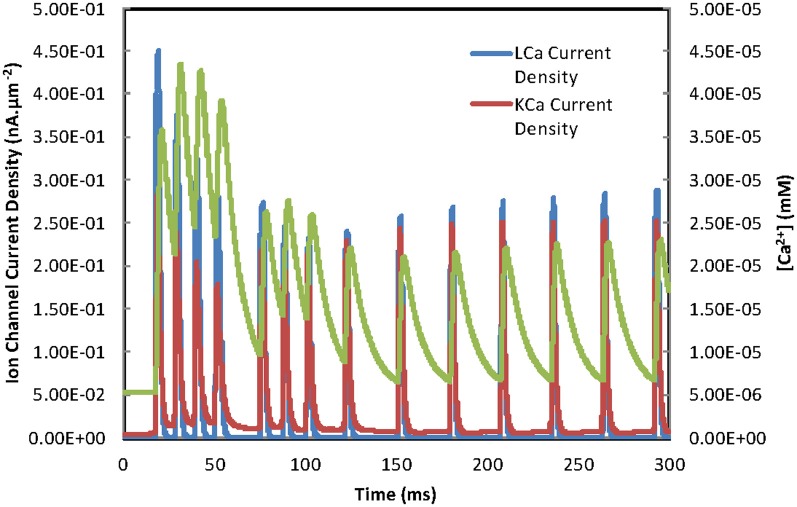
**Time dependence of calcium mechanisms during burst firing.** A range of variables, L-type calcium channel current, IKCa channel current and concentration of the calcium-pool, plotted as a function of time are taken from the simulation in Figure 8C. The change from burst firing to repetitive firing results from reduced current maxima of the L-type calcium channels (see text for further details).

**Figure 10:**
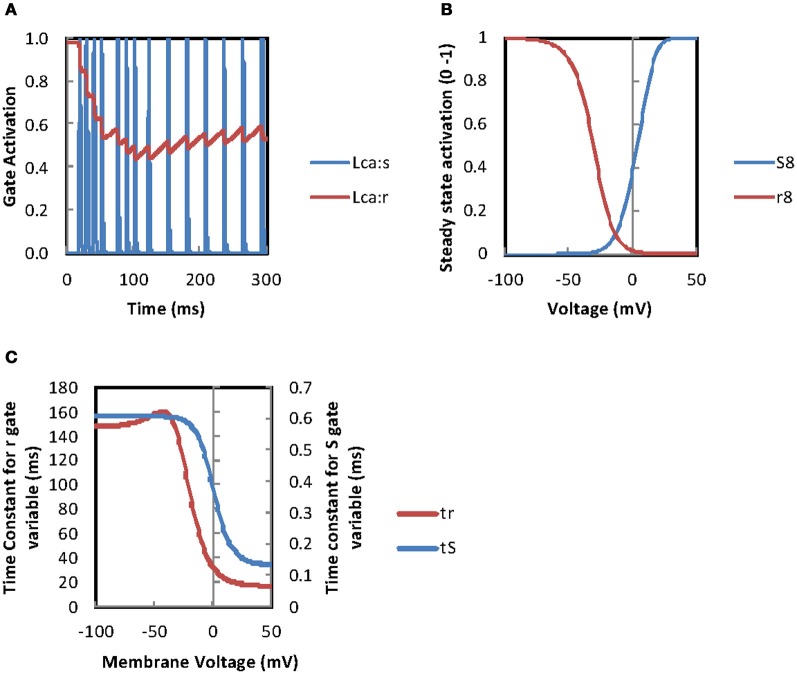
**L-type calcium channel gate parameters during burst firing. (A)** Plotting activation of the “s” and “r” L-type calcium channel gate variables from the simulation in Figure 8C shows that the “r” gate activation decays to around 50% explaining the decay in L-type calcium channel current density maxima in Figure 9. (B) The steady state activation of the “s” and “r” gate variables for the Bhalla and Bower L-type calcium channel plotted against membrane potential. **(C)** Plotting the time constants for the “s” and “r” gates against membrane voltage for the Bhalla and Bower (1993) L-type calcium channel shows long time constants for the “r” variable. This may prevent the activation levels recovering in panel **A** (see text for discussion).

In an effort to understand why the continuous current injection simulation (Figures 8C,D) produces a rhythmic firing pattern after 120 ms rather than further episodes of burst firing some of the model's variables for cell 2 were investigated (Figure 9). It can be seen that there is a decay in the maximum current density through high threshold calcium channels over time while KCa channel maximum current densities are maintained. This allows the KCa channels to terminate further APs after a single AP until a recovery period has elapsed when a further AP is evoked resulting in repetitive firing rather than burst firing. Figure 10A is a plot of the gating variables “*s*” and “*r*” for the high threshold calcium channels for the simulation shown in Figures 8C,D. These plots show that the decay in the high threshold calcium channel current density maxima over time can be attributed to the partial activation of the “*r*” gate variable and cannot be attributed to the voltage dependency of the “*r*” gate variable (see Figure 10B). Plotting the time constants for the “*s*” and “*r*” gate variables (Figure 10C) reveals that the time constant (τ) for the “*r*” variable is long (range ~150 ms to 18 ms) so after initiation only partial activation is possible. Accurate measurement of the calcium channel parameters particular time constants are needed to assess the accuracy of this model.

## Discussion

Detailed models of neurons require reconstructions of cell morphology in combination with accurate estimates of the PPs. The electrotonic dimensions of the cell are a product of the PPs. Without a good estimate of these parameters the interactions between the active conductances that allow neurons to process input data in a controlled manner can only be guessed at. Detailed morphological reconstructions are rendered meaningless without the associated PPs.

This importance of PPs in producing meaningful models with accurate morphology has provided the incentive to produce accurate estimates. Several studies feature estimation of PPs by direct fitting as a means to establish their credibility (Clements and Redman, 1989; Major et al., 1994; Stuart and Spruston, 1998; Thurbon et al., 1998; Roth and Häusser, 2001).

The techniques that make up the parts of the direct fitting process have become established and in some cases even routine. The use of Neurolucida in combination with biocytin is routinely used for cell reconstruction. “Neuron” and “GENESIS” are widely used to simulate the functioning of neurons in compartmental models, but further refinement is needed before results obtained by direct fitting can be considered universally credible.

We have noted that the values we have found for *R*_*m*_ are substantially less than the findings of some researchers in other brain regions. After testing, we believe that our values are an accurate reflection of the MCs we have recorded from. As an understanding of the factors controlling the magnitude of PPs are determined further evaluation of our results will emerge. Paired sets of reconstructed morphologies with their associated PPs are invaluable to modelers, but even more important are the patterns of attributes that only become evident when many different cells from different brain regions are compared.

In this paper we assume that PPs are uniform throughout the cell. Others have found the need to include a somatic shunt resistance to fit recorded current transients (Clements and Redman, 1989). In another study, if a somatic shunt was included, a wide range of *R*_*m*_ values gave acceptable fits, while setting the shunt to zero restricted the range of PPs that produced acceptable fits (Major et al., 1994). We believe the somatic shunt was needed because of the inadequacies of microelectrode recording and can be discarded when using patch-clamp recording (Roth and Häusser, 2001).

Next we consider the problem of non-uniqueness in which a range of possible combinations of parameters give equally good fits (Roth and Häusser, 2001). We tested to see if non-uniqueness is a problem with the two recording pipette protocol (Stuart and Spruston, 1998; Roth and Häusser, 2001) by producing simulated recordings for target parameters and checking how accurately the algorithm coped with the fitting. This produced a perfect fit for all cell reconstructions, which suggests that it would not be unreasonable to assume a high degree of confidence in the direct fitting algorithm we are using. We used multiple starting points when fitting experimental recordings to avoid the risk of identifying local minima in the optimization process.

However, there are still sources of error in other parts of the protocol that need accounting for. Fixation shrinkage, pipette location as measured by inter-pipette distance, and soma morphology are spatial accuracy problems that result from difficulty in precise measurement. It is possible to improve on the scalar accuracy of the model by for example increasing “nseg” (number of nodes in a section). However, until we have the ability to improve the accuracy of the experimental estimated measurements this would assume a precision that is not justified. A refinement to the experimental method would be achieved by marking the exact location of the pipette for identification in the reconstruction process. Also, if the conversion of the Neurolucida reconstruction to a Neuron compatible morphology file could place the identified pipette locations at positions 0.5 along a section then as long as “nseg” remains an odd number the electrode would always be in precisely the right position. These two amendments would effectively remove electrode location as a source of error. In addition, reconstruction from the unfixed tissue slices would remove the effects of fixation that currently distort the reconstruction.

Extensive discussion of the error resulting from lower frequency recorded noise have been made elsewhere (Major et al., 1994; Thurbon et al., 1998; Roth and Häusser, 2001). For the recordings used to constrain the PPs in the MC models for this study, noise was filtered above 6 kHz and the cells were pharmacologically isolated to minimize spontaneous synaptic inputs. While Major et al. (1994) considered this to be an important source of error, analysis by Thurbon et al. (1998) found no significant effect, even when the noise was magnified by a factor of 10, after inserting sampled noise into simulated curves for target parameters. Roth and Häusser (2001) also came to the conclusion that signal noise was not a significant source of error when they carried out balanced resampling of the experimental recordings using a bootstrap method.

Once the PPs have been established and incorporated into a passive model, the model can be used to establish the electrotonic dimensions of the MC. Traditionally these dimensions are assessed in terms of electrotonic length but we have chosen to use neuromorphic renderings of electrotonic transformation (see “Methods”) which allow a more intuitive representation of the electrotonic dimensions. Using Neuron's “Impedance Shape” tool allows the functional measurement of impedance to correct for deviations from calculated electrotonic length (L) due to non-infinite cylinders and branching structures (Zador et al., 1995). The use of impedance also allows an assessment of frequency effects. Comparisons of the electronic transformations with unmodified reconstructions show that the apical dendrite allows for greater ease of current flow than the lateral dendrites and axon. The apical tuft, with its complex branching structure, becomes much more elongated, reducing the synaptic stimulation that it receives. This is likely to increase the required summation of multiple synaptic stimuli needed to provoke spiking in the soma. This is true across the wide range of frequencies for which input is known to activate MCs. The lateral dendrites are also seen to remain electrotonically elongated in response to output from the soma in the passive cell. The length of lateral dendrites is a consideration when looking at the ability of back propagating spikes to provoke lateral inhibition.

In our study the PPs *R*_*i*_, *C*_*m*_, and *R*_*m*_ for rat OB MCs have been fitted to dual pipette short pulse current injection recordings using the PRAXIS fitting algorithm in Neuron with detailed morphological reconstructions of the same cells from which the recordings were made. The mean values and standard deviation for the parameters were: *R*_*i*_ = 173 ± 99 Ω cm, *C*_*m*_ = 1.91 ± 0.20 μF cm^−2^ and *R*_*m*_ = 3547 ± 1934 Ω cm^2^. Compared to published figures for cells in other brain regions, the *R*_*m*_ values are very low and *C*_*m*_ values are rather high. Experiments to assess sources of error such as fixation shrinkage, inaccurate estimation of pipette location, and estimation of soma surface area from 2D outline of the fixed cell, have proved the parameter values to be both credible and accurate. The model was used to assess the electrotonic dimensions of a MC, which suggested that the apical dendrite allows a greater ease of current flow than the lateral dendrites and axon. The apical tuft is much more electrotonically elongated than would be expected for efficient transmission of input.

The Migliore model (Migliore et al., 2005) uses two MCs with simple canonical morphology and identical PPs. The ADTs consist of 20 unbranching cylinders each of which is connected by a GJ to the same cylinder component of its pair. Each ADT cylinder receives a continuous current injection. The current injection to one of the cells is started 10 ms after the other and the resulting asynchronously firing MCs rapidly synchronize across GJs. Replicating the Migliore model synchronization test protocol (Migliore et al., 2005) in our model, the spiking of two MCs becomes synchronized even more efficiently when using the reconstructed cells with the fitted PPs. This is achieved by the 100 GJs in the reconstructed cell model generating a ~42% higher coupling ratio than the Migliore model (Migliore et al., 2005). Fewer GJs did not allow synchronized firing. The cross sectional dimensions of the fibers in the ADT are the limiting factor rather than the GJ conductance. Increasing the number of gap junctions brings more of the tuft into play, while increasing the conductance needs to use the same portion of the tuft so is limited by the section of fiber in proximity to the gap junction.

Differences between the PPs of the two MCs do not affect the ability of the GJs to mediate synchronization. However, these differences in PPs are visible in the sub-threshold depolarizations (see Figure 5). Correlated spikes in MC pairs have been observed in experimental recordings from MC pairs whose ADTs were contained in the same glomerulus (Schoppa and Westbrook, 2002). However, this correlation has been assessed with cross-correlograms using 20 ms bins while the synchronization observed in our model is sub ms.

Adding four further MCs to the GJ network substantially reduces (~–23%) the coupling ratio between the two test MCs. The extra four cells and extra GJs required for networking produce a current sink that reduces coupling ratios between individual pairs. The requirement for a higher current injection to maintain firing frequency is further evidence of the current sink effect of the MC network. A consequence of this higher input is that synchronization is no longer so efficient. Tight clumping of spikes is achieved rather than complete synchronization.

One of the reasons for the lack of synchrony across the network is the difference in PPs between individual cells. The MCs in the model are from different animals and different locations within the OB. Simulations in which one of the individual PPs was fixed to a single value for all the cells demonstrated that differences in *R*_*i*_ between the cells of the model has a greater influence on synchronization than the other PPs. The following factors are emerging as a requirement for synchronization of a intraglomerular population of MCs to be achieved:

1. A greater number of lower conductance ADT GJs for the same MC coupling ratio (100 GJs per pair MCs was better than 1 or 10). This suggests that proximity of the GJs to the site of current injections improves the degree of synchronization.
2. A larger number of lower value current injections for the same total current input amplitude. The total current flow between cells in this case is not limited by the maximum conductance of individual gap junctions.
3. Similar *R*_*i*_ for all the cells. Without this the current will tend to flow in the direction of least resistance and this will not aid synchrony.

The Migliore et al. (2005) model produces oscillations for the purpose of investigating mital cell GJ synchonization by using a continuous current injection. In real MCs *in situ* there are many sources of oscillations in firing rate both intrinsic and via network interactions. The Bhalla and Bower model (1993) includes calcium channels and calcium dependent ion channels in the MCs that cause the MCs to exhibit burst firing. The burst firing characteristics of MCs have been defined by Ma and Lowe (2010) and out of a sample of 41 MCs they found 20 (49%) that exhibited spontaneous bursting properties. The Bhalla and Bower channels were fitted in a MC model to turtle MC recordings (Mori et al., 1981) which show bursting properties. The calcium channels in the Bhalla and Bower model are nominally L-type and there has been some controversy about whether L-type calcium channels are present in MCs. However, it should be noted that the available measurements of channel kinetics both then and now for high threshold calcium channels do not allow them to be differentiated into different types for the purpose of modeling. The controversy stems from the work of Isaacson and Strowbridge (1998) who investigated dendritic signaling linked to N and P/Q type calcium channels associated with neurotransmitter release. They did not investigate if L-type channels are found in other parts of the MC that are not associated with dendrodendritic inhibition. We conclude that while it is desirable that measurements of channel kinetics be made to improve the accuracy of the model, the Bhalla and Bower (1993) calcium channel mechanisms do allow us to investigate the impact of burst firing on GJ synchronization in MCs. Once these channels are included in the 6 cell model it is found that the resulting burst firing is disruptive to the synchronizing action of GJs in intra-glomerular populations of MCs. However, when a continuous current injection is applied to the cells it is observed that this disruption is temporary and synchronized firing resumes after about 120 ms. This loss of disruption is produced by the calcium channel having a very slow time constant that does not allow the channel to reactivate for a considerable period of time once it has first been activated. Slow time constants are a characteristic of calcium channels involved in burst firing. However, since the kinetics of calcium channels in MCs have not been fitted experimentally these conclusions need verification.

### Influence of other cells in the OB

MCs are grouped in intra-glomerular populations linked by ADT GJs. The network of PG cells appear to serve the purpose of suppressing activity in this population if low levels of stimuli are received from the olfactory receptor nerves, as well as being a site of some centrifugal suppression of stimuli. While the granule cells are a site of recurrent inhibition onto the lateral dendrites which slows the frequency of firing and is also involved in both lateral and centrifugal inhibition. The connected nature of this population of MCs is likely to increase depolarization of the population which will in turn increase the likelihood of the magnesium block on NMDA receptors being overcome, lengthening the period of recurrent inhibition and slowing the firing rate. However, as shown in Ma and Lowe (2010) a percentage of MCs exhibit burst firing which we have shown is strong enough to disrupt MC intra-glomerular synchrony. The purpose of this disruptive mechanism might be to allow different frequencies to prevail if cells are in a different state as suggested by Heyward et al. (2001).

### Extending the model

Extending the model to incorporate more cell types, for example periglomerular and granule cells, is an obvious progression for this network model. Simulations incorporating granule cells were tried, but they destabilized the interactions occurring with the existing range of parameters. This extension to the model was therefore postponed until such time as the basic MC network model had been sufficiently developed and refined. It will be the subject of future studies.

### Conflict of interest statement

The authors declare that the research was conducted in the absence of any commercial or financial relationships that could be construed as a potential conflict of interest.

## Footnotes

^1^It is premature at this stage to look at those output neurons that differ in size and location from the mitral cells sufficiently to be classed as tufted cells without a clear understanding of what makes them different. There is, however, evidence emerging to separate the external tufted cells from the mitral cells on functional grounds with a synchronizing oscillation for juxtaglomerular cells being attributed to them (Hayar et al., 2004). This aside, in this paper mitral cells will mean those cells whose soma lie in the mitral cell layer and whose size is reasonably uniform (give the range of total membrane area from the reconstruction). Often in the literature the term mitral/tufted cells is used because it is difficult to be certain that electrodes have been inserted in cells that are completely representative of the narrower definition of mitral cells.

^2^The NaH_2_ P0_4_ is not included in the recording solution to avoid precipitation, thus an extra 1 mM NaHC0_3_ buffer was added.
